# Supplementary material for: Mental health problems and social media exposure during COVID-19 outbreak
Source: PLoS One. 2020 Apr 16;15(4):e0231924. doi: 10.1371/journal.pone.0231924 (PMC7162477; doi:10.1371/journal.pone.0231924)
Supplement: S1 Table — (DOCX) [file pone.0231924.s001.docx]

The questionnaires were used to measure SME and depression and anxiety

**Social media exposure:**

最近一周，您从社交媒体（微博、知乎、豆瓣，公众号）获得此次新型冠状病毒肺炎的的相关信息的频率？

1. 从不 2. 偶尔 3. 有时 4. 经常 5. 总是

During the past week, how often do you were exposed to news and information about COVID-19 on social media, such as Sina weibo, Zhihu, Douban, WeChat and etc.

1. Never 2. Once in a while 3. Sometimes 4. Often 5. Very often

**Depression**

| **过去两周**  **Over the last two weeks** | **一直如此**  **All of the time** | **大部分时间**  **Most of the time** | **一半以上时间**  **More than half of the time** | **不到一半时间**  **Less than half of the time** | **有时**  **Some of the time** | **从来没有**  **At no time** |
| --- | --- | --- | --- | --- | --- | --- |
| 1我感觉生活愉快、心情好  I have felt cheerful and in good spirits | 5 | 4 | 3 | 2 | 1 | 0 |
| 2我感觉平静和放松  I have felt calm and relaxed | 5 | 4 | 3 | 2 | 1 | 0 |
| 3我感到充满活力、精力充沛  I have felt active and vigorous | 5 | 4 | 3 | 2 | 1 | 0 |
| 4我睡醒时精神焕发，感觉得到足够休息  I woke up feeling fresh and rested | 5 | 4 | 3 | 2 | 1 | 0 |
| 5我每天的生活充满了有趣的事情  My daily life has been filled with things that interest me | 5 | 4 | 3 | 2 | 1 | 0 |

Anxiety

| **过去两周**  **Over the last two weeks** | **几乎每天**  **nearly every day** | **不到一半时间**  **more than half the days** | **有几天**  **several days** | **完全不会**  **Not at all** |
| --- | --- | --- | --- | --- |
| 1感觉紧张，焦虑或急切  Feeling nervous, anxious or on edge | 3 | 2 | 1 | 0 |
| 2 不能够停止或控制担忧  Being unable to stop or control worrying | 3 | 2 | 1 | 0 |
| 3 对各种各样的事情担忧过多  Worrying too much about different things | 3 | 2 | 1 | 0 |
| 4 很难放松下来。  Having trouble relaxing | 3 | 2 | 1 | 0 |
| 5 由于不安而无法静坐。  Being so restless that it is hard to sit still | 3 | 2 | 1 | 0 |
| 6 变得容易烦恼或急躁  Becoming easily annoyed or irritable |  |  |  |  |
| 感到似乎将有可怕的事情发生而害怕  Feeling afraid, as if something awful might happen |  |  |  |  |
